# New paradigm in combination therapy of siRNA with chemotherapeutic drugs for effective cancer therapy

**DOI:** 10.1016/j.crphar.2022.100103

**Published:** 2022-04-28

**Authors:** Krishan Kumar, Varsha Rani, Mohini Mishra, Ruchi Chawla

**Affiliations:** Department of Pharmaceutical Engineering & Technology, Indian Institute of Technology (Banaras Hindu University), Varanasi, 221005, U.P., India

**Keywords:** siRNA, Chemotherapeutic drug, Combination therapy, Gene silencing, RNA interference, Apoptosis, Multidrug resistance, Cancer treatment

## Abstract

Chemotherapeutics drugs play a pivotal role in the treatment of cancer. However, many issues generate by chemotherapy drugs, including unfavorable harm to healthy cells and multidrug resistance (MDR), persist and have a negative impact on therapeutic outcomes. When compared to monotherapy, combination cancer therapy has many advantages, like improving efficacy through synergistic effects and overcoming drug resistance. Combination treatment may comprise several chemotherapeutics drugs and combinations of chemotherapeutic drugs with some other therapeutic options such as surgery or radiation. Cancer treatment that utilizes co-delivery strategies with siRNA and chemotherapeutic drugs has been shown to have highly effective antitumor effects in the treatment of many cancers. However, the highly complex mechanisms of chemotherapeutic drugs-siRNA pairs during the co-delivery process have received little attention. The ideal combination of chemotherapeutic drugs with siRNA is very crucial for producing the desirable anticancer effects that would greatly enhance therapeutic efficiency. This review puts an emphasis on the logic for choosing suitable chemotherapeutic drug-siRNA combinations, which may open the way for the co-delivery of chemotherapeutic drugs and siRNA for treating cancer in the clinic. This review summarizes recent breakthrough in the area of diverse mechanism-based chemotherapeutic drugs-siRNA combinations in cancer treatment.

## Highlights

• Chemotherapeutic drugs in combination with siRNAs.
• Overcoming multidrug resistance using the combinatorial approach.
• Use of Combinatorial therapy to promote apoptosis.
• Extracellular barriers to RNAi mediated siRNA therapy.
• Nanocarrier chemotherapeutics-siRNA co-delivery system.

## Graphical abstract

**Figure:**
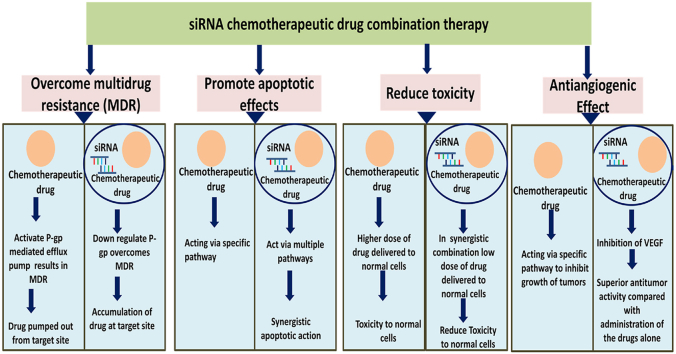

## 1 Introduction

Multiple gene mutations are responsible for the development of cancer. Cancer progression is characterized by dynamic genetic alterations and a complex system of interactions between cancer cells, which include a range of distinct cellular processes that form tumors. The complexity of signaling pathways, associated with various mechanisms to avoid programmed cell death, makes healing cancer a difficult task (Li et al., 2013). Therefore, approaches involving combination therapy have been investigated and successfully implemented in the treatment of different types of cancers. Combination treatments have been shown to be extremely successful, whether they are based on combination of multiple chemotherapeutics or chemotherapeutics combined with radiation and surgery to alter various disease pathways (Miles et al., 2002). Small interfering RNA (siRNA) has emerged as a potential therapeutic tool. The use of siRNA in the form of a drug demands great care and attention. siRNA, a category of the exogenous oligonucleotide, could even prompt the innate immune system, and may not be as safe as initially assumed. Interestingly, the immunogenicity of siRNA can be reduced through chemical modifications or improved sequence design (Judge and MacLachlan, 2008). siRNA therapeutics is safe and effective treatment option for variety of diseases, particularly for which there are not any specific and very effective treatments available, such as cancers with significant benefits such as high specificity, many possible routes of administration and ability to silence multiple genes at the same time (Chen and Zhaori, 2011). Co-delivery of chemotherapeutic drugs and small interfering RNA (siRNA) combination for cancer treatment has been identified as an encouraging treatment approach that has sparked interest around the world.

Chemotherapy is one of the most popular therapeutic approaches used in cancer treatment today. The first chemotherapeutic agent given for the treatment for cancer was a compound called nitrogen mustard during World War II, which was later categorized as DNA alkylating agent. Following the success of the former in the treatment of lymphoma, several other chemotherapeutic agents with similar mechanisms that interfere with cellular or metabolic processes have been also identified (Khan et al., 2012). The chemotherapeutic drugs damage healthy cells, cause multidrug resistance (MDR) and have a negative impact on therapeutic outcomes (Liu et al., 2013). Most therapeutic agents are non-selective, which causes significant harm to normal cells. Poor permeability of these agents lead to poor penetration across the tumorous tissue, increasing the dose and the frequency of dosing for these agents (Hu et al., 2016). Multidrug resistance (MDR) is primarily caused by two mechanisms: drug efflux pumps on the cell membrane and anti-apoptotic mechanisms. Cancer cells develop MDR due to presence of enhanced efflux pumps which results in lower intracellular concentration of medication, which ultimately leads to sub-therapeutic effect. The anti-apoptotic mechanism, on the other hand, helps them to survive the anti-tumor effect of chemotherapy drugs due activation of anti-apoptotic proteins, such as Bcl-2, Bcl-xL, Mcl-1, and BI-1, and decreasing expression of pro-apoptotic molecules (Bad, Bax, Bak, Bim, Puma, Noxa Apaf1) (Rouble et al., 2013). The efficacy of chemotherapeutic drugs is limited by these physiological mechanisms and thus warrant newer approaches (Gandhi et al., 2014).

**Fig. 1:**
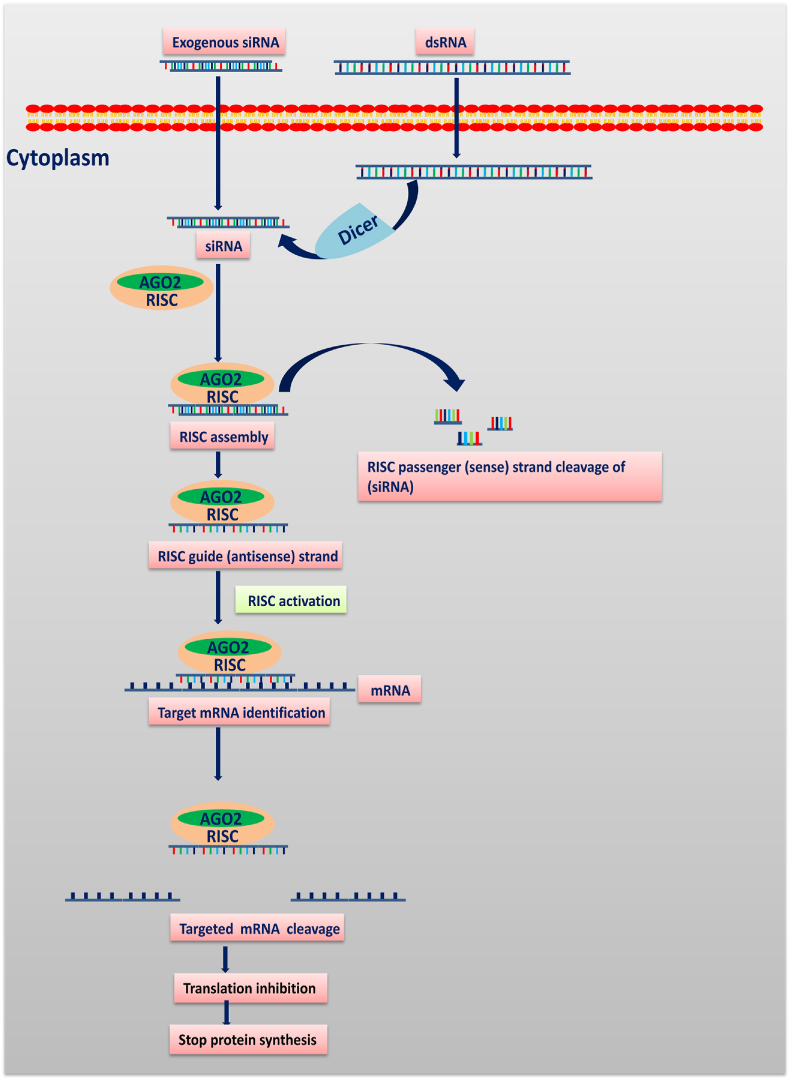
**Process of siRNA mediated RNA interference:** The siRNA pathway starts with the Dicer enzyme complex cleaving long double-stranded RNA (dsRNA) into siRNA or introducing exogenous siRNA into the cytoplasm. Then siRNAs are bound to Argonaute 2 (AGO2) and the RNA-induced silencing complex (RISC). If the RNA duplex packed onto RISC has an ideal complementarity sequence, AGO2 cleaves the passenger (sense) strand, resulting in active RISC containing the guide (antisense) strand. The siRNA guide strand detects target sites for direct mRNA cleavage (brought out via catalytic domain of AGO2), which results in translation inhibition that further blocks protein synthesis (de Fougerolles et al., 2007).

RNA interference (RNAi) technology as shown in Table 1 is being investigated as a possible strategy for developing highly targeted RNA-based gene-silencing treatments. Small interfering RNA (siRNA) based on the RNA interference (RNAi) technology is emerging as a potent interventional strategy in cancer management. RNA (siRNA) can efficiently shut down cellular pathways by knocking down genes, paving the way for better therapeutics for diseases caused by abnormal gene expression (Filleur et al., 2003). RNA interference (RNAi) is a vital biological mechanism that silences pathological proteins endogenously. They inhibit expression of genes by binding to target messenger RNA (mRNA) and disrupt translation once they reach the cytoplasmic RNA-induced silencing complex (RISC). This approach has proven to be effective in genomics, target validation, and remedial treatment. The term siRNA refers to a class of duplex RNAs (20–24 nucleotides long) that are identified by the enzyme DICER, which cause gene-specific cleavage by complementarily pairing with mRNA. A simplified description of siRNA's molecular mechanism is described in Fig. 1 (Tekade et al., 2016). Although siRNAs have remarkable anticancer activity, when injected intravenously, siRNA chains have a short half-life in the blood due to intravascular degradation by ribonuclease (RNase) enzymes. Rapid systemic clearance of siRNA renally, low specificity for the preferred tissue, and inadequate cellular uptake has been observed when administered intravenously, reducing the efficacy of therapy (Chen and Zhaori, 2011). In the last few decades, RNAi therapeutics have made substantial progress and Nano therapeutics mediated approach not only helps to protect loaded RNAi from in vivo breakdown but also allows intracellular delivery at the target site, avoiding off-target silencing. Nanoparticles (NPs) are efficient drug carriers, which allow loading of both hydrophilic and hydrophobic drugs with effective drug encapsulation easing internalization by tumor tissues, thereby increasing therapeutic efficacy, and reducing the side effects. Various Nano platforms have found application for the encapsulation and delivery of genetic material safely and bio compatibly (Xiao et al., 2017). The nanoscale materials like siRNA (approximately 7.5 ​nm in length and 2 ​nm in diameter (Schroeder et al., 2010)) face the challenge of degradation in the in vivo condition, in addition to transfer across cells/tissues/organs as a result of their large size (∼13 ​kDa) and negative charge (Subhan and Torchilin, 2019), (Lee et al., 2013). These issues are solved by chemical changes to the RNA backbone and the use of nano-sized carriers (Wang et al., 2010). To enhance siRNA delivery, electrostatic modifications of surface were used to prepare multilayer nanoparticles. These changes have been used to improve therapeutic stability in vivo, insert cell-specific targeting ligands, and encourage controlled release (Shmueli et al., 2010).

**Table 1:** RNA interference -based approaches used for cancer therapy.

RNAi approaches	Description	Target mechanism
siRNA (Small interfering RNA)	Double-stranded RNA (dsRNA) oligonucleotides (21–25 base pair) that are either generated artificially or are endogenous dsRNA products.	siRNA is bound by RNA-induced silencing complex (RISC) and unwound it into single-stranded strands that binds to the complementary mRNA sequence. The pairing causes cleavage at the target sequence, and the cleaved mRNA is degraded and prevented from being translation (Mahmoodi Chalbatani et al., 2019)
(shRNA (Short hairpin RNA	shRNA is made up of a 19–20 base pair RNA sequence with a short hairpin loop of 4–11 nucleic acid.	The shRNA-plasmid enters the cell, integrates with the host nuclear DNA, and generates pre shRNAs that are transferred into the cytoplasm, where the shRNA cleaves the target mRNA with the help of the dicer complex. (Acharya, 2019).
lncRNA (Long non-coding RNAs)	lncRNA are made up of over 200 nucleotides. However, they lack the ability to code for proteins.	These RNAs may be involved in chromatin remodelling, transcription regulation, and RNA processing in the nucleus, but in the cytoplasm ​they usually perform their functions by interacting with mRNAs and proteins (Pecero et al., 2019), (Ratti et al., 2020).
miRNA (Micro RNA)	miRNAs are single-stranded, non-coding RNA oligonucleotides (20–25 base pairs). Multiple siRNAs can be created from a single miRNA transcript.	They target mRNAs with base-pair recognition and initiate mRNA degradation, which reduces the levels of the respective protein (Valencia-Sanchez et al., 2006).
ASO (Antisense oligonucleotide)	ASO are synthesised, single-stranded nucleic acids with short (18–30 nucleotide) sequences that are related to a cellular RNA target.	Base-pairing reactions that disrupt or correct pre-mRNA splicing and processing in order to suppress translation or induce the degradation of targeted mRNAs (Crooke et al., 2021).
Aptamers	Aptamers are single-stranded nucleic acids (DNA/RNA) that have a high affinity and specificity for their targets. They have random sequence of oligonucleotides. In other words, they are antibody-like nucleic acid analogues.	They bind with great affinity and specificity to target proteins, acting through three-dimensional structures, recognizing secondary structures of lncRNAs, and interfering with RNA-protein interactions (Fathi Dizaji, 2020).

In recent times, the use of chemotherapeutic drugs in combination with siRNAs has opened a new window for targeted management of cancer and is attracting the interest of researchers worldwide. The dual therapy of siRNA-based oncogene interference and chemotherapeutic drug-mediated destruction of tumor cells not only reverse chemotherapeutic drug resistance and effectively reduce unwanted damage to healthy cells, but also show synergistic/combinatorial anticancer effect (Gandhi et al., 2014), (Li et al., 2016). The combination of siRNA with chemotherapeutic drugs is highly beneficial because multiple mechanisms and regulatory proteins associated with tumor cell growth, development, metastatic spread and drug resistance can be manipulated at the same time to achieve better therapeutic efficacy (Babu et al., 2017). For example, knocking down proteins involved in MDR such as p-glycoprotein which act as efflux pumps and Bcl-2 (anti-apoptotic gene) which is responsible for developing cancer by preventing release of mitochondrial cytochrome *c* and regulates survival of cancer cells over apoptosis through the non-pump mechanism, could improve chemotherapeutic efficacy (Ma et al., 2013), (Ray, 2020). As a result, drug/gene co-delivery is emerging as a handy tool for targeting the cells at molecular level. In this technique, pairing of siRNA-chemotherapy is a crucial step in designing an integrated combination therapy.

This review puts an emphasis for choosing suitable chemotherapeutic drug-siRNA combinations, which may open the way for the co-delivery of chemotherapeutic drugs and siRNA for treating cancer in the clinic. This review discusses significant advancements in the development of various mechanism-based chemotherapeutic drug-siRNA combinations for co-delivery in cancer treatment. This article is expected to provide formulation scientists with an update on the progress made toward the development of chemotherapeutic-siRNA-based combinations.

## 2 The rationale for selecting chemotherapeutic drug-siRNA pair for treatment of cancer

**Fig. 2:**
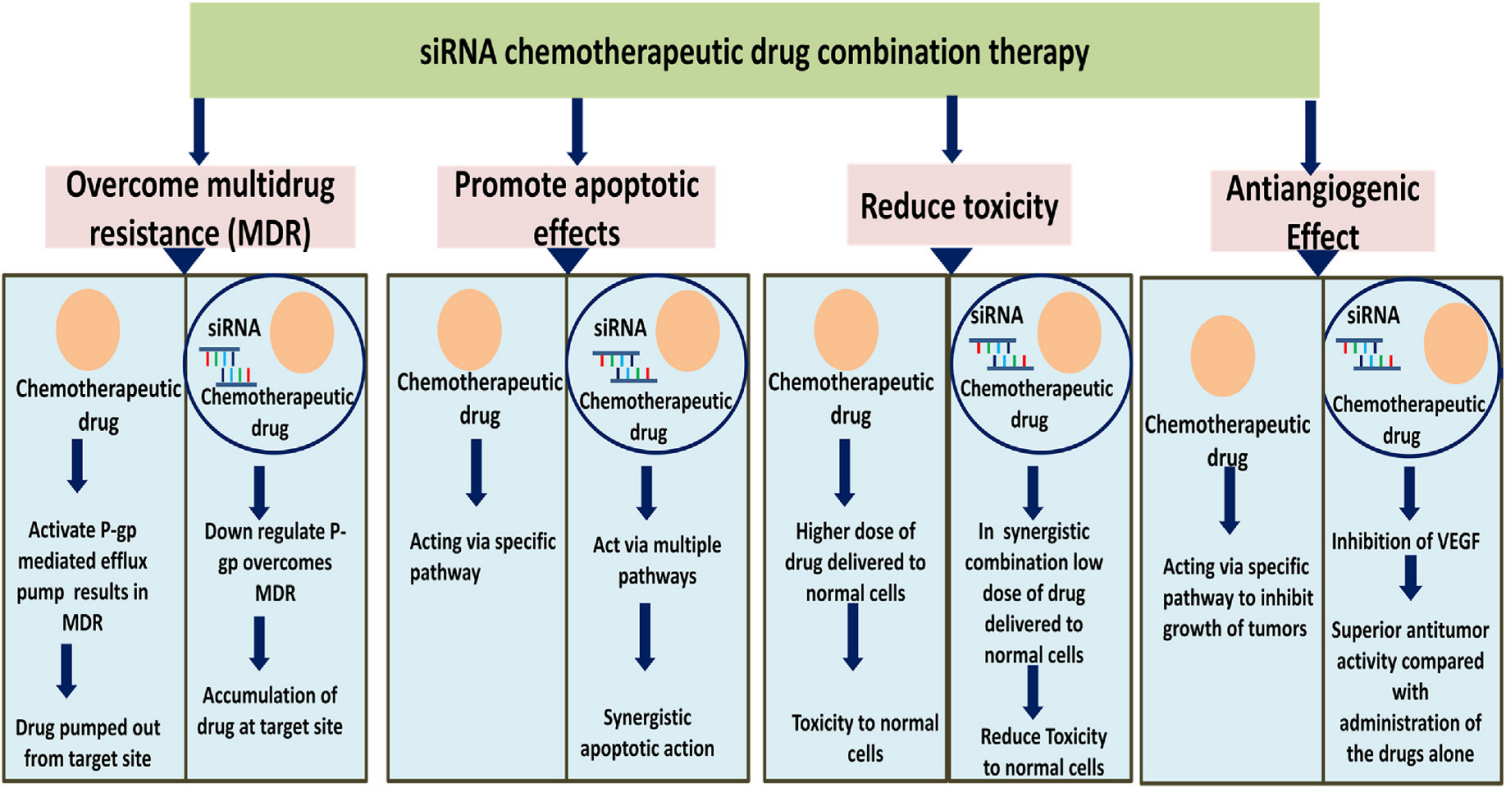
Advantages of combination therapy of siRNA with chemotherapeutic drug for safe and targeted treatment of cancer.

Pairing of chemotherapeutic drugs and siRNAs is a crucial step in the success of the therapy and there are various strategies used for the selection of the pair. In addition, understanding of the basic principle for suitable pair selection can provide many-fold increase in antitumor activity with the reduction of the dose. So either co-delivery systems using chemotherapy drug-siRNA pairs with same mechanism of action such as anti-proliferation or anti-angiogenesis effect on a two-dimensional scale are used or co-delivery systems with different mechanisms of action such as the combination of anti-angiogenesis effect, or anti-proliferation effect, or reversal of MDR, or inhibition of metastasis (invasion) can be selected (Wang et al., 2017). The benefits of combining siRNA therapy with chemotherapeutic drugs for cancer targeting have been demonstrated in Fig. 2.

Oncology research is currently focused on identifying and targeting specific genetic changes in cancerous cells, probably leading to cancer and understanding of genetic mutations in cancerous guides selection of chemotherapeutic drugs alongwith a development of advanced non-traditional gene-based therapeutics (Oh and Park, 2009). The non-traditional siRNA based therapy helps to knock down genes that are probably responsible for abnormal proliferation of cancerous cells directly or indirectly. Further, siRNA-based gene silencing targets cells that are otherwise not targeted by drugs, antibodies, and proteins (Soutschek et al., 2004). Sufficient data from in-vivo and in-vitro studies have shown that siRNA-mediated gene silencing could significantly inhibit abnormal cancerous cell proliferation and also, increase the sensitivity to chemotherapeutic drugs (de Fougerolles et al., 2007). Further, mutated genes can be targeted with therapeutic agents that not only kill the tumor tissues but also protect the normal tissues from collateral damage (Bharat et al., 2020). RNA interference is currently a widely used tool for identifying and targeting them. White et al. used one-well/one-gene high-throughput screening platform combined with genome-wide siRNA library to systematically investigate the molecular basis of NCI–H1155 cancerous cell sensitivities (Whitehurst et al., 2007). . Studies have shown that certain experimental groups responded to paclitaxel concentration that was 10,000 times lower than otherwise required for the response. Furthermore, significant reduction in cell number was responsible for cell death instead of a transient halt in proliferation. This study demonstrates that the combined effect of chemotherapy and RNAi technology might be a useful approach for significantly increasing the therapeutic potential of chemotherapeutics in a synergistic way. Notably, many recent studies show that the combination of a chemotherapeutic drug and siRNA has excellent anticancer effect when compared to either the chemotherapeutic drug or siRNA alone (Yang et al., 2014; Yin et al., 2014; Beh et al., 2009; Chen et al., 2009). It has been demonstrated that suppressing the expression of B-cell lymphoma2 (Bcl-2), an essential anti-apoptotic protein, inhibits tumor growth. Zheng et al. observed that the combination of docetaxel and Bcl-2 siRNA down-regulated the expression of Bcl-2 genes and increase the anticancer potential that consequently inhibited tumor growth in a murine xenograft model of MCF-7 breast cancer cells compared with either docetaxel or siRNA used alone (Zheng et al., 2013).

## 3 Significance for chemotherapeutic drugs and siRNA combination mechanisms

Combination therapies based on chemotherapeutic agents and RNAi technology drastically enhance cancer treatment efficacy by targeting distinct cellular signal pathways, which enhance the genetic hurdles for cancerous cells mutations. As a result, this strategic approach can retard the tumor adaptation system thereby improving therapeutic efficacy. In order to combine chemotherapy and RNAi technology, it is necessary to select a suitable gene target. The following genes have been targeted using this combinatorial therapy: (1) genes which play a role in cancer drug resistance (e.g., a gene encoding P-gp) (2) genes responsible for survival of tumors (e.g., gene encoding Bcl-2) (3) genes expressed selectively in cancerous tissues (e.g., gene encoding VEGF) (Xiao et al., 2017).

### 3.1 Overcoming multidrug resistance using the combinatorial approach

A single drug therapy is generally not effective in treating cancer because of the heterogeneity and complexity of the mechanisms involved in the regulation of the progression of the diseased state. Owing to its complex pathophysiology, cancer is prone to multidrug resistance (MDR), which is characterized by concomitant resistance to more than one drug (Saraswathy and Gong, 2014). Decreased drug influx, increased drug efflux, activation of cytochrome P450 (detoxifying systems), activation of DNA repair and blockage of apoptosis (decreased ceramide levels), overexpression of ATP binding cassette (ABC) transporters (MDR1 (ABCB1), MRP1 (ABCC1), and ABCG2) are the contributing factors responsible for the development of MDR (Meng et al., 2013a). MDR turns out to be a significant hurdle in the treatment of cancer, and combinatorial therapy can be an effective tool to overcome it to a great extent (Babu et al., 2017). Administration of multiple therapeutic agents having different cellular targets, co-administration of more than one anticancer agent differing in their mechanism of action, and co-administration of MDR modulators with anticancer agents are some promising approaches well explored for treating patients with MDR (Saraswathy and Gong, 2014), (Meng et al., 2013a). Synergistic effects exhibited by co-delivery of siRNA and anticancer agents loaded in nanocarriers have gained popularity in recent times. Xu et al. developed inhalable siRNA nanoparticles embedded with porous microparticles to overcome MDR in lung cancer candidates. Initially, they prepared siRNA-loaded chitosan nanoparticles via ionic gelation method; the siRNA-loaded nanoparticles were further co-loaded in poly-L-lactide porous microparticles having doxorubicin hydrochloride. The research group reported that the nanoparticles embedded with porous microparticulate formulation showed greater aerodynamic performance with sustained release of doxorubicin that led to accentuated anticancer efficacy in drug-resistant cells as compared to free doxorubicin and siRNA free nanoparticles (Xu et al., 2018). Donmez and Gunduz studied the effect of selectively down regulating the P-gp/MDR1 mRNA to resensitize the breast cancer cells that were reported to be doxorubicin-resistant. The effect of silencing the gene expression was determined by qPCR analysis; Donmez and Gunduz reported 90% gene silencing and 70% resensitization of the doxorubicin-resistant MCF-7 breast cancer cells towards doxorubicin (D ö nmez and G ü nd ü z, 2011). Yang et al. targeted the MDR1 gene and studied the effect of gene silencing in doxorubicin-resistant human hepatocellular carcinoma Bel-7402/ADM cells. RT-PCR was done to study the expression of MDR1 mRNA, while expression of P-glycoprotein was studied by western blotting. Successful silencing of MDR1 mRNA and P-glycoprotein expression in doxorubicin-resistant human hepatocellular carcinoma Bel-7402/ADM cells and reversal of MDR to doxorubicin in the xenograft tumor model was observed (YANG et al., 2016). Zhao et al. investigated the combinatorial therapy for the delivery of IKBKE targeted siRNA and cabazitaxel and studied its effect in triple-negative breast cancer. The research group formulated a nano complex fabricated with hyaluronic acid (meant to target CD44) for the co-delivery and reported enhanced cellular uptake with increased antitumor activity in the triple-negative breast cancer mouse model (Zhao, Li, Liu, Jain, Patel, Cheng).

### 3.2 Use of Combinatorial therapy to promote apoptosis

Drug resistance in cancer patients could also be attributed to reduced apoptosis (many anticancer therapeutic agents act through induction of apoptosis in cancer cells, hence reduced apoptosis upholds drug resistance) in cancer cells which in turn reduces the drug potency and restriction in its full use. Reduction of apoptosis enables survival of cancer cells and their accentuated resistance towards cellular stress (DNA damage, nutrient deprivation, or hypoxia), leads to a progression of cancer. Transformation of the apoptotic signaling pathway could be counted for the resistance of cancer cells towards chemotherapy, accentuated expression of anti-apoptotic genes (B-cell CLL/lymphoma 2 (BCL2), BCL2-like 1 (BCLXL), Livin/ML-IAP/KIAP), X-linked inhibition of apoptosis, and survivin represent the members of the inhibitor of apoptosis protein family (Saraswathy and Gong, 2014), (KUNZE et al., 2012). BCL2 and BCLXL prevent the release of cytochrome C from mitochondria of cells followed by caspase activation and thus hamper apoptosis while X-linked inhibitor of apoptosis directly binds to and thus inhibits caspases and survivin tends to increase the stability and activity of X-linked inhibitor of apoptosis (KUNZE et al., 2012). Livin gene is known to show a limited expression pattern, particularly in certain cancerous cells (melanoma or HeLa cervical carcinoma cells); with no or to a lesser extent in most normal tissues, livin is supposed to hinder the process of apoptosis by inhibition of caspases-3 and caspases-9 (Crnkovic-Mertens et al., 2003). Therefore, it can be concluded that accentuated resistance of cancerous cells towards apoptosis is a hallmark of cancerous cells and can be exploited for the development of therapeutic approaches to treat cancer. Scientists have well explored the potentials of conjugating siRNA with anticancer drugs to potentiate anticancer therapy. Kunze et al. studied the inhibition of antiapoptotic genes to enhance the efficacy of chemotherapy in bladder cancer cells through siRNA. They inhibited the genes of inhibitor of apoptosis protein family viz., BCL2, BCLXL, XLAP, and survivin using siRNAs in bladder cancer cells (EJ28 and J82 BCa cells). Subsequently, the cells were treated with cisplatin or mitomycin C after 24 ​h. The research group reported resensitization of EJ28 and J82 ​cells towards the therapeutic drugs cisplatin and mitomycin C with successful inhibition of targeted antiapoptotic genes (KUNZE et al., 2012). Likewise, another research group, Mertens et al. has reported siRNA mediated silencing of livin gene and successful induction of apoptosis in tumor cells (Crnkovic-Mertens et al., 2003). Tekedereli et al. formulated a nanoliposomal formulation containing siRNA targeting the BCL-2 gene and studied its effect in estrogen receptor (−) MDA-MB-231 and estrogen receptor (+) MCF-7 breast tumor xenograft models. They reported that intravenous administration of nanoliposomal formulation of siRNA (0.15 ​mg siRNA/kg) twice a week showed promising antitumor activity and suppression of growth of tumor in both the xenograft models of breast cancer. In addition to this, they also studied the synergistic effect of nanoliposomal formulation and antitumor drug doxorubicin. They found a significant increase in the efficacy of the doxorubicin when administered with nanoliposomal formulation of siRNA in both the models (MDA-MB-231and MCF-7) owing to improved apoptosis because of the silencing of the BCL-2 gene (Tekedereli et al., 2013). The p53 gene is a tumor suppressor gene, and mutations in the p53 gene lead to loss of its aptness as a tumor suppressor gene. Zhu and his research team investigated the effect of silencing the p53 gene by siRNA in 5637 and T24 human bladder cancer cell lines and reported significant inhibition of proliferation and viability of cancer cells owing to induction of cell cycle arrest in the G2 phase and apoptosis. They also evaluated the synergistic effect of siRNA-mediated silencing with the chemotherapeutic drug cisplatin and reported improved sensitivity of cancer cell lines towards cisplatin (Bin Zhu et al., 2013). Zou et al. developed a copolymer-based nanocarrier for the co-delivery of siRNA and doxorubicin and studied its effect on apoptosis in ovarian cancer cells. They developed folate conjugated poly (ethylene glycol), poly (ethylene imine), and poly (ε-caprolactone) based cationic micelles targeting the BCL-2 gene and reported a significant enhancement in apoptosis skov-3 ​cells (Zou et al., 2012). c-FLIP is reported to inhibit apoptosis and caspase 8 activation and is a potential target in cancer treatment. Longley et al. studied the apoptotic response of c-FLIP on the chemotherapies (5-fluorouracil, irinotecan, and oxaliplatin) in colorectal cancer. They reported a significant enhancement in the chemotherapy-induced apoptosis upon downregulation of c-FLIP with siRNA (Longley et al., 2006). Another research group studied the synergistic effect of siRNA targeting VEGF and Gemcitabine monophosphate administered via lipid/calcium/phosphate-based nanoparticle formulation. The therapeutic potential of the formulation was evaluated in orthotopic and subcutaneous xenograft models of NSCLC and reported a remarkable increase in apoptosis of tumor cells, decrease in tumor cell proliferation and noteworthy reduction of tumor microvessel density (Zhang et al., 2013).

### 3.3 Immunotherapy using combination of chemotherapeutic drug with siRNA

Cancer cells are supposed to vanquish the immune system of the patient, and inadequacy of chemotherapy, radiation, and surgery add to the distress of the patients. At the genetic level, many genes play a vital role in protecting cells from cancer, and any mutation in such genes acts as a setback in the management of cancer. Now it is well established that genes related to immune response could be a potential target, and reversal of immunosuppression mediated by cancer cells, could be an efficient approach in the treatment of cancer (Farkona et al., 2016). The approval of sipuleucel-T in the year 2010 for the treatment of prostate cancer, ipilimumab antibody (anti-cytotoxic T lymphocyte-associated protein 4) in the year 2011 (SL et al., 2011), and anti-programmed cell death protein 1 antibodies in the year 2014 (S. P and JP, 2015) have provided a significant boost to exploration of immunotherapy for the treatment of cancer. Adoptive cellular immunotherapy, cancer vaccines, blockage of an immune checkpoint, oncolytic viruses, and administration of recombinant proteins or antibodies are some of the strategies employed under immunotherapy in the treatment of cancer. Combined use of chemotherapy and immunotherapy can provide synergistic effect. Chen et al. developed a nano assembly loaded with an acid activatable prodrug of doxorubicin and siRNA and studied its effect on the reversal of immunosuppression. They reported a marked increase in the tumor-infiltrating T lymphocytes and ameliorated expression of interferon-γ by the PD-1 targeted siRNA and a significant boost in the immunogenic cell death effect of doxorubicin (Chen et al.). A research paper published in biomaterials studied the immunotherapeutic approach for treatment of cancer in tumor-associated macrophages and breast cancer cells. The paper demonstrated delivery of vascular endothelial growth factor and placental growth factor targeted siRNA via polyethylene glycol-based nanoparticles and reported efficient silencing of both the targeted genes and reversal of tumor microenvironment from pro-oncogenic to antitumoral (Wang et al., 2021). Li studied the effect of targeting TPP-1 peptide on the interaction of PD-1/PD-L1. It was found that targeting TPP-1 peptide significantly reduced the inhibitory effect of PD-L1 in T cells and reactivated T cells. They postulated that TPP-1 peptide could serve as a potential substitute to antibodies in tumor immunotherapy (Li et al., 2018). Similarly, Zhao et al. studied the combinatorial effect of administering immune checkpoint PD-1 targeted siRNA and pimozide in melanoma cells via attenuated Salmonella. Marked induction of apoptosis and immune response was observed, and targeting of PD-1 inhibition could serve as a potential strategy in the treatment of melanoma (Zhao et al.).

### 3.4 Combinatorial therapy for antiangiogenic functions

Tumors develop new blood vessels with the support of angiogenic molecules to meet their nutritional and oxygen needs for proliferation. For example, inhibiting vascular endothelial growth factor (VEGF) activity resulted in the down-regulation of several cancer-causing factors such as endothelial cell proliferation, angiogenesis, and tumorigenesis (Mitchell, 2013). Combination of VEGF siRNA and anticancer drugs has been reported to have excellent antitumor activity as compared to administration of drugs alone. VEGF siRNA-chemo resistant gene combinations have also shown to effectively inhibit angiogenesis (Tekade et al., 2016)**.** Several experiments have shown that, in addition to its role in angiogenesis, VEGF can inhibit cell apoptosis by stimulating specific kinases or signaling pathways, such as phosphoinositide 3-OH kinase, p44 protein kinase, c-jun-NH2-kinase, the PKB pathway, and Bcl-2. Sometimes, the existence of VEGF in the tumor environment can interfere with the delivery of chemotherapeutic drugs due to increase in the permeability of blood vessels. Furthermore, due to interstitial tumor hypertension, drugs could be effluxed out of the tumor cells, resulting in chemotherapy resistance. Therefore, VEGF downregulation could enhance the effect of chemotherapeutics, and anti-VEGF siRNA can be used to boost the efficacy of chemotherapeutic drugs (Wu et al., 2017). This was demonstrated for VEGF siRNA, and GEM monophosphate (GMP) entrapped in lipid/calcium/phosphate nanoparticles (LCPs), named as (GMP ​+ ​VEGF)-LCP-AA. When compared to monotherapy with VEGF siRNA and GMP, the (GMP ​+ ​VEGF)-LCP-AA reduced VEGF mRNA expression levels in cancer cells, inhibited tumor vasculature formation, and induced cancer cell apoptosis by a large margin with fewer side effects in H460 tumor cells (Zhang et al., 2013). VEGF siRNA treatment may have minimal side effects as compared to other anti-VEGF drugs, such as mAbs and small molecule TKIs, which have significant adverse symptoms (bleeding, neutropenia, hypertension, skin problems, and diarrhea).

## 4 Carrier systems for co-delivery of chemotherapeutic agents and siRNA

**Fig. 3:**
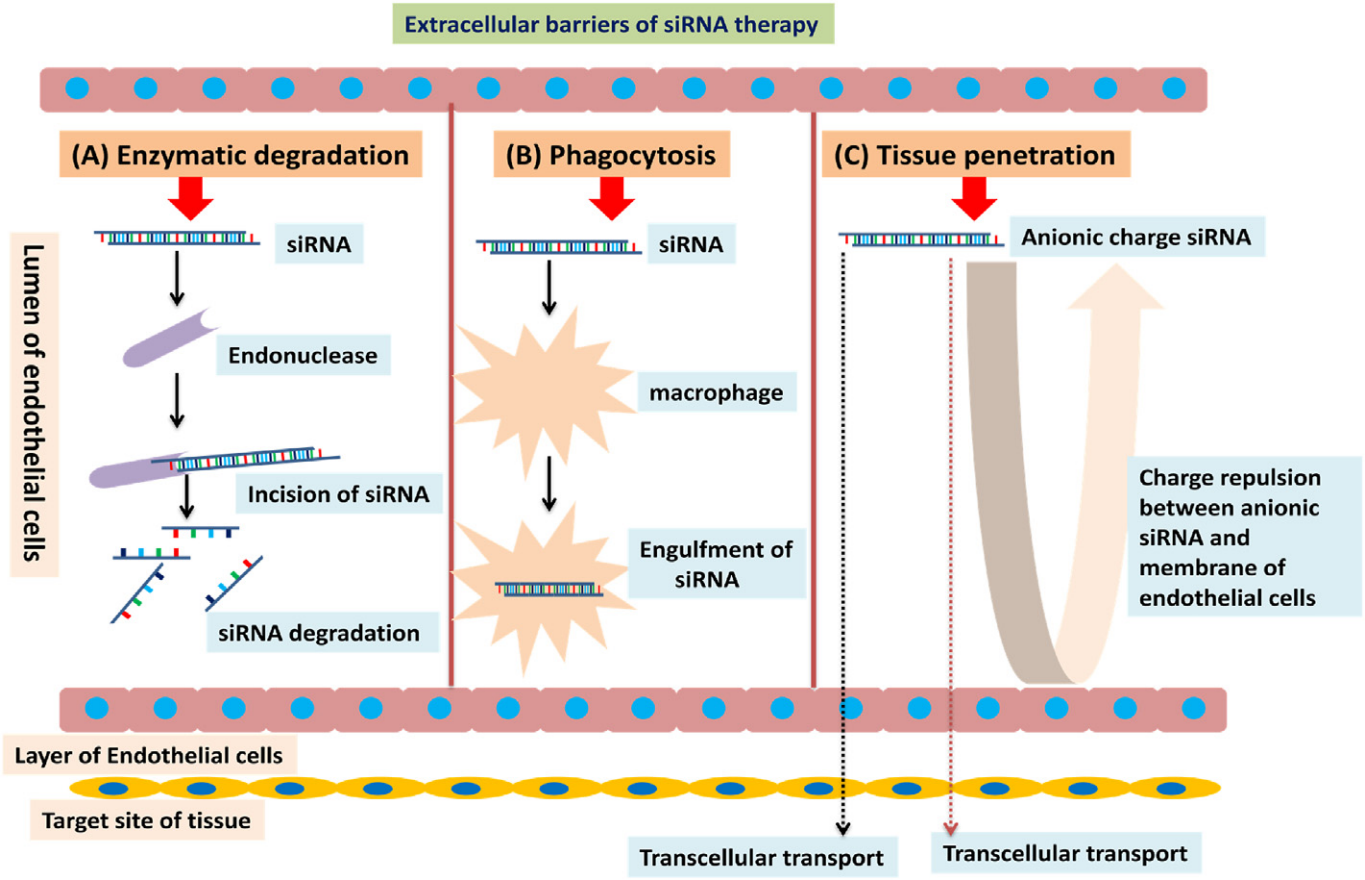
Extracellular barriers to RNAi mediated siRNA therapy **(A) Enzymatic degradation**: Endonuclease degrades siRNA in blood circulation **(B) Phagocytosis**: The phagocyte cells (macrophages) removes siRNA from blood circulation via clearance through liver, lungs, and spleen **(C) Tissue penetration:** Repulsion interactions (due to anionic charge) between siRNA and plasma membrane of endothelial cells prevent internalization of siRNA into cells, only transported by transcellular and paracellular transport mechanisms.

Nucleic acid-based drugs require sufficient cellular transfection efficiency, which can only be achieved with the use of suitable delivery platforms. Nucleic acids, including siRNAs, are water-soluble and negatively charged so it is difficult to deliver them through the cell membrane. Furthermore, they must escape in the intact form from endosomes and lysosomes before being delivered to the site of action to exert their effect. Another challenge is protecting them against degradation by the endogenous enzymes and media along with the formulation excipients (Lam et al., 2012). The extracellular barriers to RNAi-mediated siRNA therapy are described in Fig. 3 and these barriers have been exploited by the researchers for the delivery of nucleic-acid based molecules. In current discussion, two types of delivery techniques: viral vectors-based and non-viral-based delivery techniques have been discussed.

Virus-based delivery typically uses retroviruses, lentiviruses, adenoviruses as delivery vectors. Although virus-based delivery systems have a high transfection efficiency, they frequently have negative side effects, most notably the stimulation of immune response and toxic cellular effects. Because of the numerous drawbacks of virus-based delivery vehicles, there has been increased interest in the usefulness of non-viral-based delivery techniques that use nanoparticles as a delivery vehicle (Subhan and Torchilin, 2020). siRNA delivery via nanosystems is expected to protect the siRNA from breakdown while delivery to the site of action ensuring precision and accuracy of delivery.

The use of nanoparticles as a carrier for siRNA delivery have the several benefits: (1) To overcome barriers, particle size is favorable for siRNA delivery (2) They are not immunogenic because they are inert (3) they can augment interferon production and natural killer (NK) cell activity, resulting in antitumor activity and increase therapeutic efficacy (4) They have a longer circulation time, which allows them to much more effectively permeate and accumulate in tumor cells (5) they can be easily imaged and tracked. On the other hand, they have some drawbacks like low water solubility and limited bioaccumulation. However, these issues can be solved by choosing the right polymers. As a result, the benefits outweigh the drawbacks (Tatiparti et al., 2017).

### 4.1 Nanoplatforms for the co-delivery of chemotherapeutic Drugs-siRNA

To overcome the issues related to delivery of siRNA, several bioengineered nanoparticles (NPs) have been investigated and optimized. Some bioengineered siRNA conjugated nanocarriers which have been studied are lipidic NPs, polymeric NPs, inorganic NPs, and RNA-based NPS. Physico-chemical properties of nanocarriers such as size, shape, surface chemistry, and ligand-based targeting of the nanocarriers can affect biodistribution, cell-specific internalization and stability of these carriers.

#### 4.1.1 Influence of size and shape of nanoparticles

The cellular uptake and tumor accumulation via endocytosis are primarily determined by the shape and size of siRNA conjugated nanoparticles. A study demonstrated efficient cellular uptake and gene silencing along with prolonged circulation of siRNA loaded in micellar nanoparticles (MNPs) 90/siRNA with higher retention in tumor cells MDA-MB-231 murine xenograft model as compared to MNP-90/siRNA. In contrast, larger particles showed rapid clearance from the circulation with low gene silencing efficacy and antitumor efficacy (Liang et al., 2015). Similarly, TiO2 (TiO2-PEG) NPs modified PLGA nanoparticles has shown the size dependent cellular uptake of siRNA molecules, 300 ​nm NPs undergoes clathrin-mediated endocytosis efficiently than 100 ​nm and 200 ​nm NPs into NCI–H292 ​cells (Sabourian et al., 2020). Gold nanoparticle (GNP) GNPs are easily internalized by HeLa cells and significantly increases the cellular uptake rate of 50 ​nm sized GNPs as compared to 20 ​nm in MCF-7 and MDA-MB-231 tumor cell lines because of higher interacted surface area with cell membrane receptors resulting in engulfing of nanoparticle by the cells (Chen et al., 2020).

In addition, silicon-based nanoparticles demonstrated shape-dependent distribution of discoidal particles which showed higher penetration in organs than quasi-hemispherical and cylindrical shapes (Decuzzi et al., 2010). Likewise, co-polymer coated mesoporous silica nanoparticles(MSNs) (sphere and rod shaped) tagged with fluorescein isothiocyanate (FITC) and rhodamine B isothiocyanate (RITC) for quantification through imaging indicated that rod shaped MSNs have shown more cellular uptake than sphere shaped MSNs (Longley et al., 2006; Zhang et al., 2013). Furthermore, internalization efficiency of the gold nanoparticles was influenced by the shape of siRNA nanoparticles. Lower cellular uptake was shown by gold nanorods as compared with spherical shape which indicated decreased cellular uptake with increase of aspect ratio. HeLa cells were treated with various shapes of gold nanoparticles (hollow gold nanoshells (HGNS), hollow gold nanocages (HGNC), and gold nanorods (GNR)). GNR and HGN showed least knockdown with Green fluorescent protein (GFP) as analyzed by dark field microscopy and flow cytometry (Morgan et al., 2019). Similarly, 50 ​nm spheres (221 ​± ​34 AS per NP) to 40 ​nm stars (251 ​± ​41 AS per NP) with similar surface densities of overall RNA (0.13 molecules nm−2) indicated 1.6 times higher cellular uptake efficiency of spherical NPs in comparison to nanostars at 24 ​h (Yue et al., 2017). Nanospherical and nanoneedle Poly (lactic-co-glycolic acid) polyethylene glycol nanoparticles (PLGA-PEG) nanoparticle undergoes cell internalization via endocytosis but needle shaped PLGA-PEG nanoparticle induces cytotoxicity in the cell lines followed by lysosome disruption and eventually DNA fragmentation and cell death (Zhang et al., 2017).

#### 4.1.2 Effect of surface charge of nanoparticles carrying siRNA on delivery

Surface charge (negative or positive) on the nanoparticles influence the pharmacokinetic and biodistribution pattern of these carriers. The negatively charged nanoparticles undergo electrostatic repulsion by the cells leadings to the non-delivery of contents into the cells as compared to positively charged nanoparticles (Velikonja et al., 2013), (Li and Gu, 1021). The cationic nanoparticles reduce the membrane integrity and thus facilitates internalization and cellular uptake of nanoparticle inside the cell. Whereas, the anionic nanoparticles interact more with phagocytic cells than positively charged nanoparticles which leads to cytotoxicity (Foroozandeh and Aziz, 2018).

Although positively charged and negatively charged particle has capability to reach target site by following various targeting mechanism for delivery of siRNA. Both kinds of nanoparticle have some advantages and disadvantages, so developing a suitable nanocarrier is major challenge. Some literature has shown cationic nanocarrier as good for delivering siRNA with higher cellular uptake and cytotoxicity (de Lima and Pedroso, 2003). Some have shown the potential of anionic nanocarrier to serve as a good vehicle with higher transfection efficiency but rapidly cleared from the systemic circulation (Patil et al., 2004). To deal with this contradictory statement, surface charge density becomes an important aspect rather than surface charge only (Weiss et al., 2021). Surface medication becomes a necessary tool to cater such issues that enables improved stability, transfection efficiency, cellular uptake with reduced cytotoxicity and systemic clearance. However, anionic nanoparticle has been modified into a stable anionic lipid-siRNA complexes as a delivery system with minimal cytotoxicity by using a divalent cationic bridging agent such as calcium. A novel formulation has been developed to improve toxicity and efficient siRNA delivery. Anionic liposomes1,2-dioleoyl-sn-glycero-3-phospho-(1′-rac-glycerol)-1,2-dioleoyl-sn-glycerol- phosphoethanolamine (DOPG/DOPE) were complexed with siRNA using calcium ion bridges to prepare anionic lipoplexes (Kapoor and Burgess, 2012) (Tagalakis et al., 2014).

Studies reported the stearylamine (positively charged) decorated Poly Lactic acid—Poly Ethylene Glycol (PLA-PEG) nanoparticles showed higher internalization compared to PLA-PEG nanoparticles in HeLa cells indicated that positively charged nanoparticles possess better cellular uptake capability than negatively charged nanoparticle with less cytotoxicity. The cationic nanoparticles undergoes micropinocytosis and clathrin/caveolin mediated endocytosis mechanism whereas, anionic nanoparticles undergoes clathrin-dependent/caveolin-independent endocytosis for their cellular uptake (SL et al., 2011; S. P and JP, 2015).

In contrast, some authors have reported the lower cellular uptake and induced toxicity inspite of greater capacity of positively charged nanoparticles to electrostatically interact with negatively charged phospholipidic cellular membrane due to agglomeration of cationic nanoparticles. A comprehensive study on cationic carbon nanoparticles by assessing the in vitro and in vivo lung toxicity on THP-1-derived macrophages by evaluating zeta potential value and surface charge density clearly reveals that the surface charge density of a cationic carbon NP is a relevant descriptor of cytotoxicity rather than zeta potential. Higher Charge density of carbon nanoparticle has shown the high viability loss of THP-1-derived macrophages (Sun et al., 2019). Similarly, gold nanoparticles functionalized with ammonium bromide showed the pronounced cytotoxicity compared to alkyl sodium carboxylate, or poly(ethylene glycol) (PEG)-terminated gold nanoparticles while no cell death was seen after exposure to the carboxylated or PEG-modified gold nanoparticles (Gallud et al., 2019).

The immunogenicity, carcinogenicity, and inflammation restricted clinical efficacy of viral vectors. The non-viral gene carriers like Lipidic nanoparticles, polymeric nanoparticles, and inorganic nanoparticles are a good choice due to less immunogenicity with tunable size, surface and charge properties for delivering siRNA. The surface charge of non-viral vectors provide a safe and biocompatible strategy for siRNA delivery with enzymatic protection, efficient cellular uptake (Han et al., 2012). Various studies have shown that cationic polymers are prefrerred choice as a nanocarrier with better stability and ease of preparation. Cationic liposomes prepared from polyethyleneimine (PEI), poly(L-lysine) (PLL), and their various derivatives primarily act as non-viral delivery systems conjugated with siRNA to form nanoparticles act as good vehicle for transferring genes (10 folds increase in transfection efficiency) to the cell due to higher internalization capability mediated via reduced cell membrane integrity, micropinocytosis or clathrin-dependent and caveolin-independent pathways along with less cytotoxicity (Li et al., 2018; Zhao et al.). It is believed that gold nanoparticles with reversible charges enable the escape of nucleic acids from endosomes/lysosomes, thus facilitating gene delivery (Lee et al., 2009). A pH-responsive charged-reversible polymeric nanoparticle polyethylenimine (PEI)/poly (allylamine hydrochloride)-citraconic anhydride (PAH-Cit)/chitosan coated gold nanoparticles (PEI/PAH-Cit/AuNP-CS) loaded with showed no cytotoxicity against HeLa and MCF-7R cells suggested that, pH sensitive polymer PAH-Cit facilitates siRNA release (79%) via amide hydrolysis in acidic environment within late endosome and lysosome with better stabilization of siRNA and protection from enzymatic degradation (Han et al., 2012). PLGA nanoparticle can also be used for encapsulating smaller and larger (20 bp-3Kbps) siRNA molecule and incorporation of polyethylenimine (PEI) within the polymeric PLGA matrix can improves the retention and encapsulation of anionic siRNA molecules efficiently (Patil and Panyam, 2009).

#### 4.1.3 Surface chemistry of nanoparticles carrying siRNA

Polyplex is a polymeric system consists of condensed complexed gene or siRNA interacted through electrostatic interactions between cationic groups of the polymer and the negatively charged nucleic acids. Rapid degradation and poor cellular uptake and inability of transfection are the major challenges that can be overcome by synthesizing a polyplex with polyethylene glycol (PEG)-modified l-arginine oligo(-alkylaminosiloxane) grafted with poly(ethyleneimine) (PEI) for siRNA delivery. The altered surface chemistry provides stability, facilitating cellular uptake and minimizing cytotoxicity (Lu et al., 2019).

Surface chemistry has significant impact on pharmacokinetics and tumor delivery. Studies have shown that 20 ​kDa polyethylene glycol (PEG) and 20 ​kDa Poly(2-(methacryloyloxy) ethyl phophorylcholine) (PMPC) block protein absorption in vitro with higher stability in presence of salt or heparin with 5-fold higher circulation half-life as compared to 5 ​kDa PEG. Further, zwitterionic PMPC-based polyplexes showed in vivo silencing of luciferase with 3-fold higher cell uptake as compared to PEG (5 ​kDa) polyplexes. These experiments showed that zwitterionic polyplexes with high molecular weights drastically enhanced pharmacokinetics and tumor uptake (Jackson et al., 2017). Inorganic nanoparticles (metallic and non-carbon sources based particles like gold (Au) NPs, metal salts, quantum dots, super-paramagnetic iron oxide NPs, 2D-nanomaterials, and mesoporous silica NPs) (Liu et al., 2021) possess efficient loading capacity of siRNA due to higher surface area to volume ratio, the latter being attached via direct conjugation or non-covalent interactions. Additionally, optical and physical characteristics of inorganic nanoparticles aid in the visualization of siRNA delivered into cells or tissues (Jiang et al., 2016). Lee H et al. prepared self-assembled six single-stranded DNA fragments and six double-stranded siRNAs to create DNA/siRNA tetrahedral nanoparticles using simple conjugation surface chemistry for targeted delivery to the various cancerous cells (Lee et al., 2012). Surface chemistry plays a vital role in delivery of siRNA from charged nanoparticles as the ionic interactions pose inhibition to cellular uptake and non-specific aggregation of nanoparticles on interaction with protein and gene leading to reduced efficiency of delivery and increased clearance by mononuclear phagocyte system. surface coating of bioengineered nanoparticles by poly(ethylene glycol) (PEG) and hydration shields nonspecific interaction of nanoparticles with biomolecules (Kozielski et al., 2013). Structurally nanocarriers with amphiphilic character (hydrophobic and hydrophilic) can stick to membrane bilayer and can easily be absorbed. Some studies have shown that presence of long hydrophobic chains facilitate better interaction and cellular internalization as compared to single chains CTAB of (Cetyl-Trimethyl-Ammonium Bromide) and DTAB (Dodecyl-Trimethyl-Ammonium Bromide) adsorbed on the surface of nanocarriers that facilitates improved cellular uptake compared to un modified nanocarriers (Peetla et al., 2009).

Surface modification of nanocarriers using covalent and non-covalent approaches can effectively increase the biocompatibility and facilitates active and passive cellular uptake drug and siRNA. Specific protocol needed to be followed to achieve surface modification with desired characteristics: addition of larger molecule could lead to change in size and 3D conformation of nanocarriers. The conjugation of molecule on surface of nanocarriers delivering siRNA conjugation density and ligand orientation are an important aspect to influence cellular uptake. Thus, modification in surface chemistry of nanocarriers can yield smart platforms for treatment of cancer by incorporating therapeutic agents to provide controlled release therapy at specific site and decreases the toxicity (Sanit à et al., 2020).

#### 4.1.4 pH of nanoparticles carrying siRNA

The study suggested that siRNA loaded lipidic nanoparticles (LNP) are found stable and potent in the gastrointestinal (GI) tract at pH 1.2with “fasting” pepsin and bile salts concentration (Ball et al., 1038). Additionally, LNP showed lower activity in mucin coated Caco-2 ​cells that is improved by using PEG like polymer coated lipid nanoparticle (Chen et al., 2020; Decuzzi et al., 2010). The ideal characteristic for delivery of siRNA from nanocarrier is stability of the carrier and siRNAat physiological pH like gastric pH, endosomal pH. The co-localization experiments of labeled siRNAs loaded in Polyamine phosphate NCs (PANs) polymeric nanocarriers have shown stability of nanocarrier at pH 7 to 9 and it gets disassembled at the low endosomal pH to release siRNA.into cytosol due to stability of PANs from pH 7 to 9 and its instability at pH greater than 9 and lower than 6 (Andreozzi et al., 2017). Similarly, methoxy poly(ethylene glycol)-poly(histidine)-poly(sulfadimethoxine) (mPEG-PHis-PSD, shorten as PHD), a novel triblock copolymer comprises a drug delivery system with pH-induced charge-reversal characteristics, and proton sponge effect showed efficient delivery of small interface RNA (siRNA) siPLK1 for silencing of polo-like kinase (PLK1) into the cytoplasm for lung cancer treatment (Shi et al., 2018). These reported studies suggested that release of siRNA is also influenced by acting pH of the nanocarriers loaded with siRNA. The co-administration of chemotherapy regimens and siRNA has gained significant attention due to the increased anti - tumor activity over single dose. In this study, a pH-responsive chitosan-based prodrug vector for the co-delivery of doxorubicin (DOX) and Bcl-2 siRNA was designed. Glycyrrhetinic acid receptor-mediated endocytosis favoured the deposition of manufactured nanoparticles in hepatoma cells. In 10 ​h, the cumulative release amount of entrapped DOX and siRNA was 90.2 percent and 81.3 percent, respectively. More impressively, this nanoplatform may effectively combine gene- and chemotherapies in vivo, resulting in a significantly higher tumour inhibitory rate (88.0%). These findings show a paradigm for the co-delivery of multiple drugs in a single formulation, which could aid in the delivery of therapeutics and has a lot of promise in combinational clinical testing (Yan et al., 2020).

#### 4.1.5 Cellular barriers and endosomal escape of nanoparticles carrying siRNA

The siRNA delivery undergoes rapid enzymatic degradation with limited translocation across cellular barriers, and endosomal release is the major challenge for siRNA therapy. Desirable gene delivery nanocarriers must have low toxicity and high transfection efficiency across the various cellular extracellular and intracellular barriers. The intracellular barriers and extracellular barriers comprise of biological (epithelial cells, endothelial cells, endosome membrane, cytosol, blood-brain barriers and plasma membrane etc.), chemical (physiological pH and enzymatic degradation, Antibodies and opsonization reaction), and physical barriers (Mucous membrane, skin and keratinized epithelium). The main extracellular barriers faced by siRNA to arrive at site of action are endogenous nuclease, renal elimination, biological membrane impermeability, reticuloendothelial system entrapment, opsonization, plasma protein sequestration and endothelial crossing. The intracellular barriers such as endosomal trap, cytosol and off target effects also influenced the release of siRNA from nanocarriers (Sajid et al., 2020). These barriers restricts the delivery of siRNA, depending on the target organ and route of administration of siRNA delivery to reach their intended targets in the cytoplasm and to exert their gene silencing activity. Further, the presence of phagocytes, macrophages, airborne bacteria, virus and toxic particles hampers the delivery of siRNA-loaded nanoparticles to the lungs than other body organs. Surface modification of nanoparticles can be done to decrease the interaction of siRNA-conjugated nanoparticles with phagocytic cells, macrophages, airborne bacteria and toxic particles. Intracellular barriers lead to inaccurate delivery, endosomal escape or inadequate packing of siRNA conjugated nanoparticles. Endocytosis is the main mechanism for the delivery of siRNA to desired site of action that are prevented by extracellular and intracellular barriers through phagocytosis, reticuloendothelial uptake of nanoparticle and their release at random site different from site of action and later excreted by kidney (Itani and Al Faraj, 2019). However, live cell microscopy by using cytosolic galectin-9 as a membrane disrupting sensor that represents endosomal escape of cholesterol-conjugated siRNA and release of ligand-conjugated siRNA from vesicles to enhance target knockdown up to ∼47-fold in tumor cells (Du Rietz et al., 2020). Janus based nanopieces are rod-shaped nanoparticles formed by bundles of Janus base nanotubes (JBNTs) with RNA cargoes incorporated inside via noncovalent interactions of small molecules consisting of a base component mimicking DNA bases and an amino acid side chain can efficiently enter into cells via macropinocytosis indicating much better endosomal escape ability than lipid nanoparticles (Lee et al., 2021).

Moreover, endocytosis is a major challenge for siRNA delivery to the targeted site. Furtherreticuloendothelial system (RES) transports the siRNA conjugated nanoparticles RES rich organs (spleen and liver) eliminating them from the general circulation of body, recognizing them as unwanted elements leading to accumulation of siRNA in liver and spleen instead of the target organ. Incorporation of polymers like poly lactic-*co*-glycolic acid (PLGA), Polyethylenimine (PEI) have the capacity to increase endosomal escape efficiency and cellular uptake. Stimuli-responsive systems are derived from inorganic nanoparticles, lipids, and polymers are capable to deliver siRNA in a site-selective manner accomplished by endogenous stimulus (pH, redox potential, or unique enzymatic activity) to trigger the controlled release of API that primarily focusing on the principle of membrane destabilization (Lee and Thompson, 2017). The stabilization of nanocarriers can be achieved by surface-modification with PEG-conjugated vinyl ether that undergoes endosomal escape and hydrolyzed only at highly acidic endosomal environments resulted in destabilization of liposome and endosomal membrane leads to fusion of liposome and endosomes with siRNA nanocarrier to facilitates efficient release siRNA into the cytosol (Sajid et al., 2020).

#### 4.1.6 Biocompatibility and degradability of siRNA release from nanoparticles

Biomaterial degradation leads to increased biocompatibility that refers to non-toxic, non-carcinogenic, non-allergenic, non-immunogenic characteristics of the biomaterial which leads to the acceptance of the material in the body with reduced cytotoxicity, and its rate of breakdown and elimination are mediated by biological activity (Tamariz, Rios-Ram í rez). Use of reducible polymers increases biocompatibility and transfection efficacy. Poly [α-(4-aminobutyl)-l-glycolic acid] (PAGA) undergoes hydrolytic conversion into Poly(l-lysine) (PLL) analogue which exhibits high transfection with negligible toxicity and increased biocompatibility (Kozielski et al., 2013). Furthermore, the biodegradable polyethylene imines (PEIs) crosslinked with either PEG-bis-succinimidyl succinate or disulfide-containing cross-linkers have shown improved transfection efficiencies with less cytotoxicity compared to 0.8 ​kDa PEI. Similarly, arginine-grafted reducible poly(cystaminebisacrylamide-diaminohexane) polymer (ABP4.45 ​× ​10^3^ ​Da), termed as ABP polyplex, showed enhanced transfection efficiency, high cellular penetrating ability, and good nuclear localization ability (Lee and Kim, 2014). The functional nanoparticle consists of siRNA designed to achieve maximum therapeutic efficacy by reducing non-specific distribution other than target site and prevent rapid clearance of siRNA which promotes efficient delivery of siRNA to the tumor cells (Khan et al., 2019). The stealth nanocarriers layered with biocompatible polymer, most often polyethylene glycol (PEG) offer good colloidal stability in biological fluids and immune protection from the reticuloendothelial system (RES) and other immunological responses. These nanocarriers target cell specificity for siRNA loaded nanocarriers (Kolli et al., 2013). Biodegradable high molecular weight multiblock copolymer (MBC) comprise of recurrent units of low molecular weight poly (ethylene glycols) conjugated to low molecular weight cationic (L-lysine) polymer can be used for non-viral gene delivery. The MBC containing plasmid DNA is shielded from endonuclease digestion and late endosome/lysosome degradation at low pH (Bikram et al., 2004).

Poly(lactic-coglycolic) acid (PLGA) is an attractive polymer that has been regulated by the Food and Drug Administration (FDA) for the delivery of bioactive molecules because of its excellent biocompatibility, biodegradability, and excellent controlled release properties. The PLGA matrix designed to protect siRNA from nuclease degradation and allowed for a burst release of surface-localized siRNA accompanied by a triphasic controlled delivery over two months (Cun et al., 2011), (Kaur et al., 2016). PLGA copolymers have good characteristics such as decomposed into nontoxic by-product, mechanical resistance and controlled biodegradation (Mokhtarzadeh et al., 2016). PLGA based nanoparticles eliminated through biodegradation of PLGA into lactic acid and glycolic acid, although both are biocompatible and freely metabolized and finally excreted from the body through the citric acid cycle. The physicochemical characteristics of nanoparticles can cause significant differences in their metabolism processes (Wei et al., 2018). The proposed biodegradable monomethoxypoly(ethylene glycol)-poly(lactic-co-glycolic acid)-poly(l-lysine) triblock copolymers could effectively deliver siRNA into lung cancer tissues, with minimal cytotoxicity and maximum genetic transfection efficiency (Du et al., 2012).

Cationic nanoparticles are formed by use of several cationic components such as cationic initiators, cationic monomers, cationic polymers, and cationic surfactants (Ramos et al., 2014). Cationic liposomes are the nanoscale formulations containing certain cationic lipids used as synthetic carrier for nucleic acid delivery in gene silencing therapeutics (Ewert et al., 2010). Encapsulating pharmaceuticals in lipids provides structural features that are similar to those of the natural cellular bio-membrane, resulting in biocompatibility, better stability, and sustained drug release, as well as biodegradability, safety, and industrial scalability (Jagwani et al., 2020). Cationic lipids (100–300 ​nm in size) can prevent siRNA from enzymatic breakdown, extending its half-life and increasing cell absorption (Journal et al., 2018). Ionizable cationic lipids, which have a positive charge at acidic condition, have been selected over entirely charged lipids owing to low immunogenicity, reduced toxicity, and good loading efficiency. The derivatives that link up amphipathic constituents in ionizable cationic lipids are generally made of amide, ether, or ester, to which ester linkers being preferred due to its biodegradability. Patisiran ((ONPATTRO™), the very first FDA-approved siRNA preparation encapsulating in lipid nanoparticles, contains ionizable cationic lipids with ester linkages (Phyo et al., 2021). Cationic liposomes have a high biocompatibility and can pass the cell membrane to target specific genes. Commercially available cationic liposomes include LipofectamineTM 2000, OligofectamineTM, and Lipofectamine (Invitrogen) used for in-vitro transfection (Press, 2011). Cationic lipids including 1,2-dioleoyl-3- trimethylammonium propane (DOTAP), N-{1-(2,3-dioleoyloxy)propyl]-N,N,N-trimethylammonium methyl sulfate (DOTMA) and 1,2-dioleoyl-sn-glycero-3-phosphoethanolamine (DOPE) used to form cationic liposomes and complex with negatively charged deoxyribonucleic acid and siRNA, resulting in high in vitro transfection efficiency (Journal et al., 2018). Cationic lipids formulation (cationic liposomes) generally undergoes pro-apoptotic and pro-inflammatory cascades cellular pathways. The entry and interaction of cationic lipids to the negatively/neutral charged lipids in cell membranes will drastically alters the membrane characteristics that causes destabilization of membrane, and also affects the activity of membrane protein. Cationic liposome apoptosis was observed on phagocytic cells such as macrophage cell lines (RAW 264.7), mouse splenic macrophages, immature B cell line (WEHI 231), human leukemia cell line (HL-60). The apoptotic cascades induction of cationic lipids leads to several cellular events including ROS generation, Protein Kinase C (PKC) and Mitogen-activated protein (MAPK) activation, cytochrome C release and alteration in mitochondrial membrane potential that enables cells to undergoes apoptosis (Lonez et al., 2012).

### 4.2 Nanocarrier chemotherapeutics-siRNA co-delivery system

**Fig. 4:**
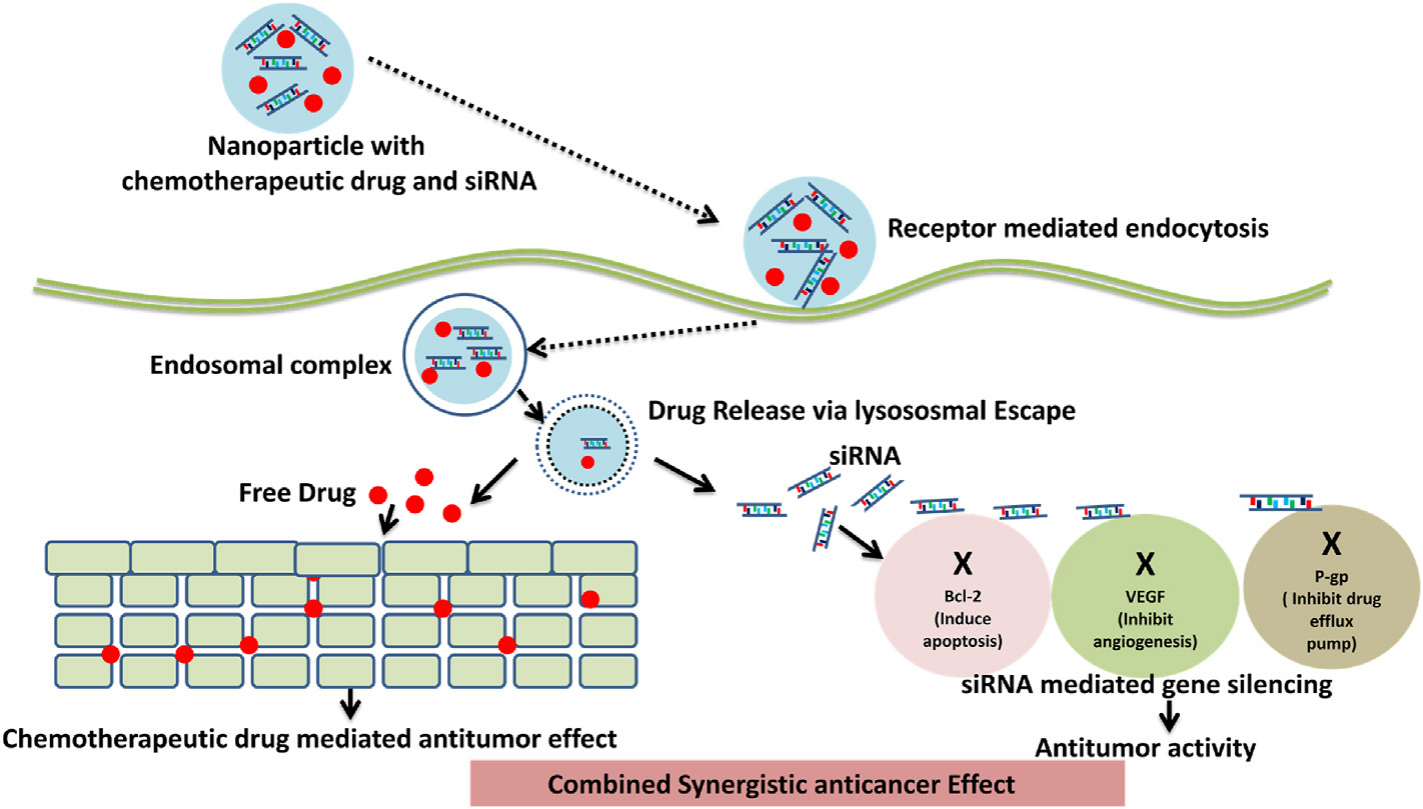
Schematic diagram of nanocarrier chemotherapeutics-siRNA co-delivery system synergistically enhance their individual anticancer effects.

Chemotherapeutic drugs are typically loaded into nanoparticles (NPs) using the “like dissolves like” phenomena while siRNA is typically encapsulated into nanoparticles using the interaction between a positively charged polymer and negatively charged siRNA (Hu et al., 2016). NPs have the capacity to co-encapsulate chemotherapeutic drugs and siRNA and, they have been used them to produce synergistic anticancer effect shown in Fig. 4 (Creixell and Peppas, 2012). Nanocarriers must enter cells and be transported through intracellular processes in order to deliver siRNA into the cytoplasm. Nanocarriers are absorbed by the cells by a variety of endocytosis mechanisms, including clathrin-based endocytosis, caveolae-based endocytosis, macropinocytosis, and additional clathrin and caveolae-independent endocytosis processes (Behzadi et al., 2017). The nanocarriers that enter the endosomes are degraded by the lysosome (pH 4.8), where the acidic pH and degradative enzymes aid in the breakdown of the nanocarriers and its payloads (e.g. siRNA). In order to prevent lysosomal breakdown, siRNA nanocarriers must escape the endosome at an initial stage. As a result of the proton sponge effect, endosome membrane fusion, pore formation, and flip flop mechanism, among other strategies, photochemical internalization has been developed to help the endosomal escape of nanocarriers (Saraswathy and Gong, 2014).

Combination treatment therapies in which a sufficient amount of siRNA and chemotherapeutic agents are delivered to the same tumor cell contribute a significant role in the treatment results. Numerous chemotherapeutic drug/siRNA co-delivery nanoparticulate systems have been developed for cancer treatment.

Liposomes are amongst the most effective drug delivery carriers, as well as co-delivery carriers for chemotherapeutics and gene agents. Recently, scientists have found success with co-delivery systems utilizing modified cationic liposomes; the most popularly used cationic lipid is 1,2-dioleoyl-3-trimethylammonium propane (DOTAP) (Skandrani et al., 2014), (Kumar and Chawla, 2021). In their study, Saad and colleagues developed a liposome-based co-delivery system that contained cationic lipids, doxorubicin, and siRNA targeting MRP1 and BCL2 mRNA (suppressors of a pump and non-pump cell resistance, respectively). The drug carrier provided excellent co-delivery technique for inducing cell death and suppressing cellular resistance in MDR lung cancerous cells (Saad et al., 2008).

Polymeric NPs have been used for the specific delivery of chemotherapeutic drugs and nucleotides. They have been employed to co-deliver chemotherapeutic drugs and siRNAs together due to their biocompatibility, stability, restricted size distribution, controlled drug release profile, and highly effective cellular absorption (Hu et al., 2010), (Wang et al., 2014). Poly (L-lactide-co-glycolide) (PLGA) though a biocompatible and non-toxic biopolymer; showed poor encapsulation for siRNAs in poly (L-lactide-co-glycolide) (PLGA). On the other hand, polyethyleneimine (PEI) demonstrated effective siRNA encapsulation efficiency (Meng et al., 2013b). Biotin-functionalized NPs made from PLGA-PEI copolymers were used to deliver phosphoglycolate phosphatase (PGP) siRNA and paclitaxel (PTX) to mammary cancer cells. These combination-based NPs

demonstrated increased anticancer activity in resistant JC (epithelial like) cancer cell line, particularly in comparison to NPs and PTX when used alone. Biotin-functionalized siRNA-PTX NPs given intravenously to tumor-bearing mice models demonstrated efficient antitumor activity (Patil et al., 2010).

Polymeric micelles, which have a nanoscale core/shell structure formed by amphiphilic block copolymers, are gaining popularity as chemotherapeutic drug/siRNA co-delivery carriers for cancer treatment. Polymeric micelles are not only effective in encapsulating water-insoluble drugs and releasing siRNA in a sustained manner, but they also have other beneficial attributes, such as a unique core/shell arrangement, passive tumor internalization via enhanced permeability and retention (EPR) effect, and additional protection of entrapped drugs from degradation and metabolism (Xiao et al., 2017). Zhu et al. developed a simple but versatile micellar system for tumor-targeted siRNA and medication co-delivery by using self-assembled copolymer (PEG-pp-PEI-PE) sensitive to matrix metalloproteinase 2 (MMP2). This system enhances cell internalization by exposing PEI to MMP2 and up-regulated tumoral MMP2 triggered delivery of the drugs with stability and in a targeted manner (Zhu et al., 2014).

Mesoporous silica nanoparticles (MSNs) are also being used extensively for drug and gene-targeted delivery applications. MSNs are non-toxic, biocompatible, and chemically stable. They are ideal for co-delivery of siRNA and chemotherapeutic drugs because of their physicochemical properties. In a recent study, functionalized MSN was developed for the co-delivery of siRNA and chemotherapeutic drugs by using PEI-PEG-modified MSNs for encapsulating epirubicin and BCl2 siRNA. BCL2 siRNA discharge from MSN was pH-dependent and the combinatorial formulation was more cytotoxic in vitro and more effective in vivo than free epirubicin and MSN carrying epirubicin, or siRNA alone. The therapeutic effect was synergistic (Babu et al., 2017).

Dendrimers are monodispersed and hyperbranched nano molecules that are composed of a single micellar molecular unit. They can be prepared in a variety of shapes and sizes, and the nature of their peripheral functional groups evaluate their physicochemical characteristics, such as drug entrapment efficiency, solubility, and toxic effects (Tekade et al., 2016). PAMAM (polyamidoamine) dendrimers are successful NPs that can co-deliver a chemotherapeutic drug as well as siRNA (Biswas et al., 2013). Low-molecular-weight water-insoluble drugs interact primarily with the PAMAM dendrimer, whereas negatively charged nucleic acids interact with cavities in PAMAM dendrimers created by surface primary amine groups via electrostatic force (Zhang et al., 2008). So, PAMAM dendrimers can be used to co-deliver hydrophobic drugs and siRNAs. Biswas et al. developed PAMAM (generation 4)-PEG-1,2 dioleoyl-sn-glycerol-3-phosphoethanolamine (G(4)-D-PEG-DOPE) triblock copolymer and then combined it with siRNA. SiRNA-delivery profile was subsequently evaluated and was improved by development of G(4)-D-PEG DOPE/PEG DOPE (1:1) micellar systems for the simultaneous delivery of chemical drug and siRNA. The dendrimer and polymeric micelles combination in a single NP can form multipurpose nanomedicine that might possibly address the issues of co-delivery of chemotherapeutic drugs and siRNAs for therapeutic purposes (Biswas et al., 2013). Multifunctional nanocarriers investigated for the co-delivery of chemotherapeutic drugs and siRNA for cancer treatment are shown in Table 2.

**Table 2:** Various nanocarrier systems for co-delivery of chemotherapeutic drugs and siRNA for tumor regression.

Nanocarrier	Chemotherapeutic drug	siRNA	Targeted gene	Targeted cell line	References
Polyethylenimine (PEI)-functionalized graphene oxide (PEI-GO)	Doxorubicin	Bcl-2 siRNA	Bcl-2	HeLa cells	Zhang et al. (2011)
Mesoporous silica nanoparticle (MSNP)	Doxorubicin	Pgp-siRNA	P-glycoprotein (Pgp)	Breast cancer cell line MCF-7/MDR cells	Meng et al. (2013b)
Trimethyl chitosan nanoparticles	Doxorubicin	HMGA-2 siRNA	HMGA-2, vimentin, and MMP9	Breast cancer cell line (MDA-MB-231)	Eivazy et al. (2017)
Nanostructured lipid carriers(NLCs)	Gefitinib and Paclitaxel	EGFR siRNA	EGFR	Human lung cancer A549, PC-9, PC-9GR, and H-1975 ​cell	Majumder and Minko (2021)
Cationic solid lipid nanoparticles	Paclitaxel	MCL1 siRNA	MCL1	KB cells	Yu et al. (2012)
Cationic Liposome	Adriamycin	siRNA RRM2	RRM2, EGFR antibody	HCC cells	Gao et al. (2013)
(FA) -conjugated polyamidoamine dendrimer	Cis-diamine platinum (CDDP)	HuR siRNA	HuR, Folate receptor-α (FRA)	H1299 lung cancer cells	Amreddy et al. (2018)
Polyethylene glycol –peptide-polyethylenimine - 1,2-dioleoyl-sn-glycero-3-phosphoethanolamine (PEG-pp-PEI-PE) nanoparticle (polymeric micelles)	Paclitaxel	BIRC5 siRNA	MMP2	A549 ​cell line, A549/T cell line	Zhu et al. (2014)
Lipid/calcium/phosphate (LCP) nanoparticle	Gemcitabine	VEGF siRNA	VEGF	H460 ​cell line	Zhang et al. (2013)
Liposome (DOTAP)	Doxorubicin	MRP1 ​+ ​BCL2 siRNA	MRP1 and BCL2 mRNA	H69AR cell line, MCF-7/AD cell line, HCT15 ​cell line	Saad et al. (2008)
Pyridylthiolterminated MSN	Doxorubicin and Cisplatin	MRP1 ​+ ​BCL2 siRNA	MRP1 and BCL2 mRNA	A549 ​cell line	Taratula et al. (2011)

## 5 Current drawbacks and challenges of chemotherapeutic drugs and siRNA co-delivery for cancer therapeutics

Gene therapy is a valuable means for the treatment of severe ailments, including cancer, despite the barriers involved in the delivery of siRNA in the body. Various factors such as fast enzymatic breakdown, short biological half-life, and low permeability across the cell membranes because of negative charge, instability, and reticuloendothelial system (RES) uptake affect delivery of siRNA. However, the development of conjugated biocompatible nanocarriers via physical and chemical treatment is possible to overcome these barriers (Reischl and Zimmer, 2009). These bioengineered nanocarriers can act as effective therapeutic agents due to less toxicity, size, charge, and chemical modification capacities that protect the siRNA loaded nanocarriers from acid/enzymatic degradation and rapid elimination from local and systemic circulation. Studies have revealed the inefficiency of RNAi therapies is primarily due to inadequate delivery of siRNA, resulting in unfavorable immune response (Sajid et al., 2020), (Gupta, Rai, Jangid, Pooja, Kulhari). So, extensive research is being conducted to obtain suitable siRNA conjugated nanocarriers having ideal characteristics like stability, biodegradability, biocompatibility that can o deliver siRNA at the target site without any immune responses (phagocytosis).

Moreover, the modification of nanocarriers using targeting moieties could be a suitable option for co-delivering siRNA and chemotherapeutic drugs limiting the expression of target protein and proliferation of cancerous cells (Rosenblum et al., 2018). The main limitation is efficient delivery of siRNA delivery are enzymatic break down, rapid renal clearance and phagocytic entrapment, but siRNA is translocated to the cytoplasm at tumor site through cellular uptake via endosomal escape mechanism (Huang and Guo, 2011). Intravenous and infusion injections are the standard route for administration siRNA loaded nanocarriers that can be mainly affected by lipophilicity and size of nanocarriers to bypass renal and hepatic clearance, reducing phagocytosis by immune cells, improving bioavailability and half-life of siRNA for prolonged period of action (Tatiparti et al., 2017). EPR (enhanced permeability and retention) effect is another phenomenon that helps to accumulate siRNA nanocarriers at tumor cells. The tumor site generally characterized by presence of leaky vasculature that enables the transcytosis of nanocarriers loaded with siRNA into the systemic circulation through these leaky vasculatures (Shi et al., 2020). The tumor vasculature, vascular permeability, structural barriers imposed by extracellular matrix and tumor cells, and intra-tumoral interstitial pressure are the primary factors responsible for EPR effects (Suk et al., 2016).

Additionally, the long half-life of the siRNA-mediated therapeutics is also pivotal to the EPR effect. The size of formulation, electrostatic nature of surface, formulation stability and lipophilicity are essential properties that protect the siRNA nanocarriers from phagocytic cells (Attia et al., 2019). The negatively charged phospholipidic bilayer cellular membrane consist of functional proteins that facilitate siRNA nanocarrier uptake and receptor endocytosis mediated through folate, transferrin, and aptamers-like receptors (Fathi Dizaji, 2020; Li et al., 2016). Furthermore, the RNA-Induced Silencing Complex (RISC) is a recent approach mainly introduced to prevent the fusion of late endosomes with the lysosomes that contain digestive enzymes (fei Xu and Wang, 2015). Another method is the use of cationic polymers to enhance the endosomolysis by their acidification, destabilization of their membranes, and rupture of the lysosomes by increasing osmotic pressure through the uptake of protons, releasing the therapeutics for efficient siRNA delivery (Bus et al., 2018). The chemical modification of siRNA by 20-O-methylation to obstruct immune response in the body after entry can deal with immunological reactions that occur in the body after encountering foreign antigens (Watts et al., 2008).

## 6 Conclusion

RNA interference (RNAi) is booming as a promising cancer treatment strategy. siRNA is a significant RNAi method that can be used to down regulate the genes responsible for drug resistance and chemotherapeutic ineffectiveness. Co-delivery increases therapeutic efficacy by providing synergistic or additive effects and by overcoming MDR. When combined with other anticancer agents, siRNA can be used to enhance therapeutic response by targeting many pathways and regulatory proteins related to cancer cell growth, metastatic spread, and drug resistance. Despite considerable progress over the past few decades, there are still a number of obstacles to be overcome before siRNA and drugs can be successfully delivered together, which significantly reduce the clinical application. Thus, combination delivery of chemotherapy drugs and siRNA as a cancer treatment therapy still requires further research. Gene-based cancer therapy has gained momentum as a result of this prospect. In the coming years, personalized therapy based on genetic makeup would be feasible, and siRNA is currently one of the most promising therapeutic approaches for the same.

## CRediT authorship contribution statement

**Krishan Kumar:** Formal analysis, Conceptualization, Project administration, Writing – original draft, Writing – review & editing, Methodology, Validation. **Varsha Rani:** Writing – original draft, Writing – review & editing. **Mohini Mishra:** Writing – original draft. **Ruchi Chawla:** Project administration, Supervision, Visualization.

## Declaration of competing interest

The authors declare that they have no known competing financial interests or personal relationships, which have, or could be perceived to have, influenced the work reported in this article.
